# Cardiometabolic comorbidities, readmission, and costs in schizophrenia and bipolar disorder: a real-world analysis

**DOI:** 10.1186/s12991-017-0133-7

**Published:** 2017-02-10

**Authors:** Christoph U. Correll, Daisy S. Ng-Mak, Dana Stafkey-Mailey, Eileen Farrelly, Krithika Rajagopalan, Antony Loebel

**Affiliations:** 1 Hofstra North Shore LIJ School of Medicine, Manhasset, NY USA; 2 The Zucker Hillside Hospital, Glen Oaks, NY USA; 3 Sunovion Pharmaceuticals Inc., 84 Waterford Dr., Marlborough, MA 01752 USA; 4 Xcenda, Palm Harbor, FL USA; 5 Sunovion Pharmaceuticals Inc., Fort Lee, NJ USA

**Keywords:** Schizophrenia, Bipolar disorder, Cardiometabolic comorbidity, Hospitalization, Healthcare costs

## Abstract

**Background:**

Serious mental illnesses are associated with increased risk of cardiometabolic comorbidities. The objective of this study was to evaluate the prevalence of cardiometabolic comorbidity and its association with hospitalization outcomes and costs among inpatients with schizophrenia or bipolar disorder.

**Methods:**

This retrospective database analysis reviewed patients with an inpatient diagnosis of schizophrenia or bipolar disorder from the Premier Perspective® Database (4/1/2010–6/30/2012). Patients were categorized into 4 cohorts based on the number of ICD-9-CM cardiometabolic comorbidities (i.e., 0, 1, 2, or 3+). Outcomes included length of stay, mortality during the index hospitalization, healthcare costs, and 30-day all-cause readmission rates.

**Results:**

Of 57,506 patients with schizophrenia, 66.1% had at least one cardiometabolic comorbidity; 39.3% had two or more comorbidities. Of 124,803 patients with bipolar disorder, 60.5% had at least one cardiometabolic comorbidity; 33.4% had two or more. Average length of stay was 8.5 (for patients with schizophrenia) and 5.2 (for patients with bipolar disorder) days. Each additional cardiometabolic comorbidity was associated with an increase in length of stay for patients with bipolar disorder (*p* < .001) but not for patients with schizophrenia. Mortality rates during the index hospitalization were 1.2% (schizophrenia) and .7% (bipolar disorder). Each additional cardiometabolic comorbidity was associated with a significant increase in mortality for patients with bipolar disorder (OR 1.218, *p* < .001), and a numerical increase in mortality for patients with schizophrenia (OR 1.014, *p* = .727). Patients with more cardiometabolic comorbidities were more likely to have a 30-day readmission (schizophrenia = 9–13%; bipolar disorder = 7–12%), and to incur higher costs (schizophrenia = $10,606–15,355; bipolar disorder = $7126–13,523) (all *p* < .01).

**Conclusions:**

Over 60% of inpatients with schizophrenia or bipolar disorder had cardiometabolic comorbidities. Greater cardiometabolic comorbidity burden was associated with an increased likelihood of readmission and higher costs among patients with schizophrenia or bipolar disorder, and an increase in length of stay and mortality for patients with bipolar disorder.

## Background

Severe and persistent mental illnesses that are often debilitating to patients, such as schizophrenia and bipolar disorder, are associated with increased physical comorbidities and mortality [1–7]. Schizophrenia is characterized by psychosis, behavioral dysfunction, and cognitive impairment and has a prevalence of approximately 1% in the United States (US) [8]. Bipolar disorder is a mood disorder characterized by intermittent periods of mania and major depression that has an approximate lifetime prevalence of 4% among adults in the US [9]. The severe psychiatric symptoms and accompanying functional disability among patients that suffer from these debilitating disorders often result in high rates of unemployment [10], incarceration [11], and suicide [12].

Compounding the psychiatric disability in schizophrenia and bipolar disorder, a growing literature suggests that physical comorbidities in this population reduce life expectancy by as much as 10–25 years and double the risk of premature mortality compared to the general population [7]. Patients with schizophrenia have 2.4 times the rate of metabolic syndrome and 2.0 times the rate of diabetes than the general population [1]. Similarly, patients with bipolar disorder have 2.0 times the rate of metabolic syndrome [2] and 1.7 times the rate of diabetes [3] than the general population. Furthermore, some evidence suggests that the prevalence of cardiometabolic risks is underestimated among patients with schizophrenia [4, 5] or bipolar disorder [9] due to under-diagnosis and under-treatment.

Complicating the inherent higher risk of cardiometabolic comorbidities in patients with schizophrenia or bipolar disorder, atypical antipsychotics, which are standard pharmacological treatment for schizophrenia and many patients with bipolar disorder, can exacerbate patients’ risk of cardiometabolic disease [13]. For example, treatment with certain atypical antipsychotics is associated with an increasing risk of developing metabolic syndrome [14, 15], diabetes [16–19], and elevated low-density lipoprotein cholesterol levels [19–21].

Hospital readmissions within 30 days post-hospital discharges (i.e., 30-day readmissions) have become an important measure of health care quality due to the high 30-day readmission rates among US Medicare beneficiaries [22]. Evidence suggests that 30-day readmissions are a significant predictor of long-term mortality [23]. In order to reduce 30-day readmission rates, the Centers for Medicare and Medicaid Services implemented the Medicare Hospital Readmissions Reduction Program which, as a penalty, reduces Medicare payments to hospitals with excess 30-day readmissions relative to the mean national readmission rates in conditions such as acute myocardial infarction and heart failure [24]. In a statistical brief summarizing the readmission trend in 2013, schizophrenia, mood disorder, and diabetes were among the top 20 conditions with the highest all-cause 30-day readmission rates [25].

While the prevalence, outcomes, and costs of cardiometabolic comorbidities in patients with schizophrenia and bipolar disorder have been examined in some outpatient studies [1–3], these variables remain relatively unexplored in hospitalized patients. In addition, although there is extensive literature regarding 30-day readmissions attributed to particular illnesses such as mental (e.g., schizophrenia) or cardiometabolic conditions (e.g., diabetes), the incremental impact of cardiometabolic conditions on readmission among patients with schizophrenia or bipolar disorder remains unknown. Therefore, the objectives of this study were to determine the prevalence of cardiometabolic comorbidities among inpatients with schizophrenia and bipolar disorder and to assess the role of incremental cardiometabolic comorbidity burden on length of stay, mortality, and healthcare costs during the initial admission. Following discharge, the study also examined the role of incremental cardiometabolic comorbidity burden on the 30-day readmission rate.

## Methods

### Study design

This retrospective observational study used administrative hospital data from the Premier Perspective Database® (Premier, Inc., Charlotte, NC, USA) during the period from April 1, 2010 to June 30, 2012. The Premier database is the largest hospital administrative database in the US and provides detailed service information from over 700 geographically dispersed hospitals and over 50 million discharges since 2000. The database contained detailed service level information, diagnostic information, hospital characteristics, and patient demographic information. The database did not include any identifiable protected health information and, pursuant to the Health Insurance Portability and Accountability Act of 1996 [26], the study did not require institutional review board waiver or approval.

### Patient selection

Patients with a primary, secondary, or admitting diagnosis of schizophrenia (International Classification of Diseases, 9th Revision, Clinical Modification [ICD-9-CM] code 295.xx) or bipolar disorder (ICD-9-CM codes 296.0, 296.1, 296.4–296.8, 301.11, or 301.13) coded during their hospitalization stays were identified between October 1, 2010 and May 31, 2012. The first such hospitalization record was designated as the patient’s index hospitalization. Patients diagnosed with both schizophrenia and bipolar disorder, patients who were less than 18-year old, or patients who were transferred from another hospital or an unknown admission source were excluded from the analysis.

### Variable definitions

Six cardiometabolic comorbidities were examined for both the patients with schizophrenia and bipolar disorder: cerebrovascular disease (ICD-9-CM 430–438.xx), coronary or ischemic heart disease (ICD-9-CM 410.xx–411.xx, 413.xx–414.xx), diabetes mellitus (ICD-9-CM 250.xx), hyperglycemia (ICD-9-CM 790.2), hyperlipidemia (ICD-9-CM 272.x), and hypertension (ICD-9-CM 401.x–405.x). Patients were categorized into 1 of 4 comorbidity cohorts based on the number of cardiometabolic comorbidity diagnoses recorded during their index hospitalization (i.e., 0, 1, 2, or 3+). In addition to examining these specific cardiometabolic comorbidities, the Charlson Comorbidity Index (CCI) was coded based on an algorithm developed for administrative data [27] using comorbidities reported during the index hospitalization as well as all inpatient or outpatient hospitalizations in the Premier database that occurred in 6 months prior to the index admission.

### Outcome variables

Outcome variables during the index hospitalization included length of stay, mortality, and costs. Following discharge from the index hospitalization, 30-day all-cause readmission rates were also examined. Length of stay in days and mortality were obtained from the discharge record for the index hospitalization. The hospitals reported both the charges for each individual service based on their charge master and the costs the hospital reported incurring to deliver the services. This analysis focused on the costs to deliver services, which were split into pharmacy and medical costs with the medical costs representing all non-pharmacy costs. All costs were adjusted to 2014 US dollars using the medical care component of the Consumer Price Index from the US Bureau of Labor Statistics [28]. The 30-day all-cause readmission rates were defined as a subsequent readmission to the same hospital for any reason within 30 days of discharge.

### Statistical analyses

Patient demographic and baseline characteristics were summarized using descriptive statistics. The relationship between cardiometabolic comorbidities and study outcomes were evaluated using multivariate statistical models controlling for the following baseline variables: age, gender, race, payer, CCI, hospital region (Midwest, Northeast, South, and West), hospital location (urban/rural), hospital type (teaching/non-teaching), and hospital bed count. For the dichotomous outcome variables, mortality and 30-day readmission, logistic regression models were used. Length of stay was treated as count data and a negative binomial regression was used. Finally, for the highly skewed cost variables, total costs, pharmacy costs, and medical costs, generalized linear models (GLMs) with a gamma distribution and log-link function were used. The statistical models were fit separately for the schizophrenia and bipolar samples. Statistical analyses were conducted using SAS version 9.4 (SAS Institute, Cary, NC, USA). All analyses were two-sided with alpha of .05.

## Results

### Patient selection

There were 118,065 patients with an inpatient hospitalization for schizophrenia. Of these, 51.3% (*n* = 60,559) were excluded for the following reasons: index hospitalization did not have the required 6-month prior observation or 1-month follow-up period; age younger than 18 years; transfer from another hospital or an unknown admission source; diagnoses of both schizophrenia and bipolar disorder. Among 229,974 patients with an inpatient hospitalization for bipolar disorder, 45.7% (*n* = 105,171) were excluded after applying the exclusion criteria as above. The final study sample included 57,506 and 124,803 inpatients with a diagnosis of schizophrenia and bipolar disorder, respectively.

### Patient characteristics

Patient characteristics are described in Table 1. The average age of patients with schizophrenia and bipolar disorder was in the mid to late 40s. The most common cardiometabolic comorbidities were hypertension (52% bipolar disorder; 57% schizophrenia), hyperlipidemia (28% bipolar disorder; 30% schizophrenia), and diabetes (22% bipolar disorder; 28% schizophrenia), with each numerically higher for patients with schizophrenia than patients with bipolar disorder. Nearly two-thirds of patients with schizophrenia (66.1%) and bipolar disorder (60.5%) had at least one cardiometabolic comorbidity, and over one-third of patients with schizophrenia (39.3%) and bipolar disorder (33.4%) had 2 or more cardiometabolic comorbidities.

**Table 1:** Demographic, clinical, and hospital facility characteristics for patients with schizophrenia and bipolar disorder

Characteristic	Schizophrenia (*N* = 57,506)	Bipolar disorder (*N* = 124,803)
*Demographics*		
Age, years (mean)	49.8	45.4
Female (%)	43.0	63.0
Race (%)		
African American	29.0	10.0
Caucasian	51.0	73.0
Hispanic	2.0	1.0
Other	18.0	15.0
Region (%)		
Midwest	24.0	24.0
Northeast	23.0	20.0
South	36.0	41.0
West	17.0	15.0
Payer (%)		
Medicaid	30.0	25.0
Medicare	51.0	35.0
Commercial/private	9.0	24.0
Self-pay	6.0	9.0
Other	5.0	6.0
*Comorbidities*		
Charlson comorbidity index (mean)	1.1	1.0
Specific cardiometabolic comorbidities (%)		
Diabetes	28.0	22.0
Hyperglycemia	2.0	2.0
Hypertension	57.0	52.0
Hyperlipidemia	30.0	28.0
Ischemic heart disease	9.0	9.0
Cerebrovascular disease	4.0	3.0
Number of cardiometabolic comorbidities (%)		
0	33.9	39.5
1	26.7	27.1
2	19.9	16.6
3+	19.4	16.8
*Hospital characteristics*		
Bed size (mean)	436	409
Urban (%)	89.0	86.0
Teaching (%)	48.0	42.0

### Outcomes

**Fig. 1:**
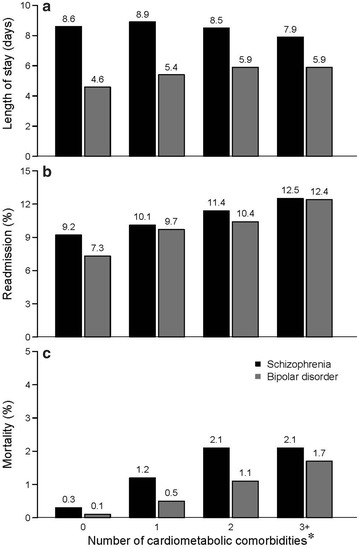
Length of stay, 30-day all-cause readmission, and hospital mortality by number of cardiometabolic comorbidities. **a** The mean length of stay was 8.5 days for overall patients with schizophrenia and 5.2 days for patients with bipolar disorder. Negative binomial regressions showed a negative association between cardiometabolic comorbidity burden with length of stay for schizophrenia (−.015; 95% CI −.024, −.007, *p* < .001), but a positive association for bipolar disorder (.029; 95% CI .024, .034, *p* < .001). **b** Overall, 11.8% of the patients with schizophrenia and 9.3% of the patients with bipolar disorder were readmitted for any reason within 30 days of discharge from the index hospitalization. For each additional cardiometabolic comorbidity, logistic regressions showed the odds of readmission increased by 3.1% (OR 1.031; 95% CI 1.001, 1.061, *p* = .042) for schizophrenia and by 6.4% (OR 1.064; 95% CI 1.041, 1.087, *p* < .001) for bipolar disorder. **c** The index hospitalization mortality rate was 1.2% for overall patients with schizophrenia and .7% for patients with bipolar disorder. In schizophrenia, cardiometabolic comorbidity was not significantly associated with mortality (OR 1.014; 95% CI .937, 1.098, *p* = .727). A Chi square test showed that patients with schizophrenia who had one or more cardiometabolic comorbidities had a higher risk of mortality compared to those with no comorbidities (1.7 vs. .3%, *p* < .001). In bipolar disorder, each additional cardiometabolic comorbidity was associated with a 21.8% increase in mortality during the index hospitalization (OR 1.218; 95% CI 1.129, 1.314, *p* < .001). A Chi square test showed that patients with bipolar disorder who had one or more cardiometabolic comorbidities had a higher risk of mortality compared to those with no comorbidities (1.45 vs. .10%, *p* < .001). * The following covariates were included in all regression analyses: age, gender, race, payer, CCI, hospital region, hospital location (urban/rural), hospital type (teaching/non-teaching), and hospital bed count

For the index hospitalization, the mean length of stay was 8.5 days for patients with schizophrenia and 5.2 days for patients with bipolar disorder. Multivariate analyses showed a negative association between cardiometabolic comorbidity burden with length of stay for schizophrenia (*p* < .001), but a positive association for bipolar disorder (*p* < .001) (see Fig. 1a).

The index hospitalization mortality rate was 1.2% for the patients with schizophrenia and .7% for the patients with bipolar disorder. For those with schizophrenia, the risk of death during the index hospitalization was not significantly associated with each additional cardiometabolic comorbidity (odds ratio [OR] 1.014; 95% confidence interval [CI] .937, 1.098, *p* = .727). Prior to correcting for baseline differences, a Chi square test showed that patients with schizophrenia who had one or more cardiometabolic comorbidities had a higher risk of mortality compared to those with no comorbidities (1.7 vs. .3%, *p* < .001). For those with bipolar disorder, the risk of death during the index hospitalization increased by 21.8% (OR 1.218; 95% CI 1.129, 1.314, *p* < .001) with each additional cardiometabolic comorbidity (Fig. 1c). Prior to correcting for baseline differences, a Chi square test showed that patients with bipolar disorder who had one or more cardiometabolic comorbidities had a higher risk of mortality compared to those with no comorbidities (1.45 vs. .10%, *p* < .001).

**Fig. 2:**
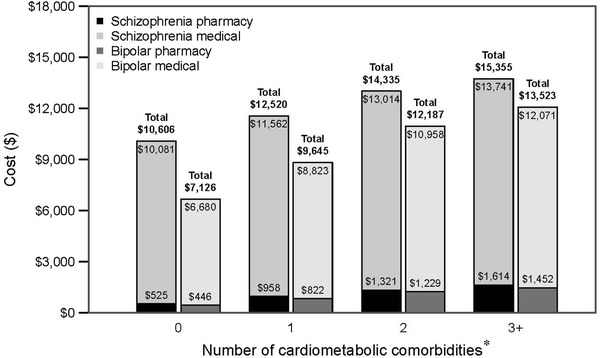
Medical, pharmacy, and total costs by number of cardiometabolic comorbidities. *Dollar figures* reflect the costs to the hospital to deliver care in 2014 dollars. For both schizophrenia and bipolar disorder, increasing cardiometabolic comorbidity was associated with increased pharmacy, medical, and total index hospitalization costs (all *p* < .001). The following covariates were included in the gamma regression analyses with a log link: age, gender, race, payer, CCI, hospital region, hospital location (urban/rural), hospital type (teaching/non-teaching), and hospital bed count. * The data are presented by number of cardiometabolic comorbidities. Overall mean total cost for patients with schizophrenia was $12,781 per patient (medical and pharmacy costs of $11,771 and $1010 per patient, respectively). Overall mean total cost for patients with bipolar disorder was $9725 per patient (medical and pharmacy costs of $8878 and $847 per patient, respectively)

 Hospitalization costs increased as the number of cardiometabolic comorbidities increased (see Fig. 2). For patients with schizophrenia, the mean total cost for the index hospitalization was $12,781 per patient (medical and pharmacy costs of $11,771 and $1010 per patient, respectively). Medical costs increased by 6.8%, pharmacy costs by 25.9%, and total costs by 8.3% (all *p* < .0001) for each additional cardiometabolic comorbidity. For patients with bipolar disorder, the mean total cost for the index hospitalization was $9725 per patient (medical and pharmacy costs of $8878 and $847 per patient, respectively). Medical costs increased by 12.3%, pharmacy costs by 26.6%, and total costs by 13.4% (all *p* < .0001) for each additional cardiometabolic comorbidity.

Within 30-days of discharge from the index hospitalization, 11.8% of the patients with schizophrenia and 9.3% of the patients with bipolar disorder were readmitted for any reason. Odds of readmission increased by 3.1% (OR 1.031; 95% confidence interval [CI] 1.001, 1.061, *p* = .042) for patients with schizophrenia and by 6.4% (OR 1.064; 95% CI 1.041, 1.087, *p* < .001) for patients with bipolar disorder (see Fig. 1b) with each additional cardiometabolic comorbidity.

## Discussion

In this large, nationally representative administrative database study of hospitalized patients with schizophrenia and bipolar disorder, cardiometabolic comorbidities were common. Over 60% of patients had ≥1 cardiometabolic comorbidity and over 30% had ≥2 cardiometabolic comorbidities. Increasing cardiometabolic comorbidity burden was associated with a significantly higher mortality rate (for bipolar disorder), and longer hospital stays (for bipolar disorder). Patients with schizophrenia appeared to have almost double the rates of mortality in comparison to patients with bipolar disorder. The average total all-cause hospitalization cost was $12,781 and $9725 per patient for schizophrenia and bipolar disorder, respectively. Each incremental cardiometabolic comorbidity was associated with an 8.3 and 13.4% increase in total hospital cost for patients with schizophrenia and bipolar disorder, respectively. While 1 in 10 schizophrenia or bipolar disorder patients had an all-cause readmission within 30-days after index hospitalization, the odds of 30-day readmission increased with each incremental cardiometabolic comorbidity.

The reported frequencies of cardiometabolic comorbidities in this study are generally consistent with those previously reported in the literature [1, 2, 15, 29, 30]. However, this study identified a higher prevalence of diabetes in patients with schizophrenia and bipolar disorder (28 and 22%, respectively) than previously reported (7–15%) [4, 6, 30–33]. This discrepancy in the reported prevalence of diabetes may be due to the study population; previous studies have typically been drawn from broader health system populations [30], clinical trial participants [4], or primary care settings [6]. Among the general population, nearly 20% of hospital stays in the US are associated with diabetes [34]. The prevalence of diabetes found among inpatients with schizophrenia (28%) or bipolar disorder (22%) in this study is therefore plausible, given that they are already considered a high-risk population for diabetes [33].

To the best of our knowledge, this is the first study to evaluate the risk of 30-day readmission among hospitalized patients with schizophrenia or bipolar disorder and its association with incremental cardiometabolic comorbidity burden. Our study showed that incremental cardiometabolic comorbidity burden was associated with a 3.1 and 6.4% increased risk of early readmission in patients with schizophrenia and bipolar disorder, respectively. A recent chart review study of 945 patients hospitalized in a psychiatric care facility found that psychiatric readmission in the following year was independently predicted by higher body mass index (BMI) [35]. The authors hypothesized that inflammation, which has been associated with both higher BMI and obesity as well as psychiatric disorders [36] may represent the link between the greater BMI and need for readmission, but research examining the mechanisms of early readmissions and cardiometabolic comorbidities is needed.

For patients with bipolar disorder, additional cardiometabolic comorbidity burden was associated with an increase in the length of stay (4.6 days for no comorbidities to 5.9 days for 3+ comorbidities). Surprisingly, increasing cardiometabolic comorbidity burden was associated with a small decrease in the length of stay among patients with schizophrenia (8.6 days for no comorbidities to 7.9 days for 3+ comorbidities). While the reasons for these differences in length of stay are not known, it is possible that bipolar disorder patients may have been more likely to receive medical assessment and/or intervention for comorbid conditions than were patients with schizophrenia in this study; alternatively it is possible that patients with schizophrenia were more likely to have medical comorbidities that were well established and known to treatment staff compared to patients with bipolar disorder.

Prior research has clearly established a link between cardiometabolic conditions and mortality in the general population [37]. The lack of statistical significance between the odds of mortality and cardiometabolic comorbidity burden in schizophrenia after correcting for demographic and hospital characteristics was unexpected; however, a univariate analysis showed a significant association with comorbidity burden and mortality. Given the small sample sizes and the rarity of mortality incidence in this dataset, it is also plausible that the potential association between mortality and cardiovascular comorbidity is underestimated. Cardiovascular disease, along with cancer and suicide, has also been established as one of the leading causes of death for patients with schizophrenia [38]. Although information about the cause of death was unavailable, confounding of the results by suicide is likely small, as all the patients were hospitalized at community hospitals and not psychiatric hospitals, indicating severity of a medical, rather than psychiatric condition at the time of admission.

Previous studies have reported increased outpatient or total costs for psychiatric patients with cardiometabolic comorbidities [39–41]. This study is unique in that it demonstrated the possible relationship between each additional cardiometabolic comorbidity and incremental costs per admission for patients with schizophrenia or bipolar disorder.

The results of this study highlight the importance of identifying optimal treatment regimens for patients with serious mental illness. Efforts should be taken to adequately monitor for and reduce the rates of cardiometabolic comorbidities in this vulnerable patient population, and perhaps consider antipsychotic therapeutic options with a limited liability for such comorbidities [13]. From a holistic approach of treating patients with serious mental illness, clinicians should coordinate care and consider a patients’ medical profile when prescribing medications. Coordinated care can also improve quality of care and patient satisfaction [42], which may have a positive effect on reducing healthcare costs through shorter hospital stays and/or reduced early readmissions. Furthermore, improved physical health may also positively impact psychiatric health outcomes [35]. In 2013, four new measures of the Healthcare Effectiveness Data and Information Set were added to assess quality of care for patients with serious mental illness; two of these four measures focus on diabetes monitoring and cardiovascular monitoring, which highlights the importance of monitoring cardiometabolic risks among this susceptible patient population [43]. Monitoring patients’ cardiometabolic profiles, consideration of these risk factors when selecting antipsychotic drug therapies, and striving to coordinate care delivery for both mental and physical symptoms may maximize patients’ outcomes.

### Limitations

The data used in this study were collected for administrative reasons rather than for research purposes. The study design precludes any determination of causal relationship between cardiometabolic comorbidities and the outcomes. The analysis was restricted to variables present in this particular database, and other factors that were not available in the database may have confounded the observed relationships. Information about the prior treatments for the mental and physical morbidities, prior hospitalizations, and reasons for death was unavailable. In particular, data on atypical antipsychotic utilization prior to hospitalization were not available, therefore it was not possible to assess the relationship between specific antipsychotic medications and cardiometabolic comorbidities, which have been previously described to vary substantially [2, 13, 20, 33].

The burden of cardiometabolic comorbidities in this study was measured using diagnostic ICD-9-CM codes of 6 disease entities. Studies that have used the more robust National Cholesterol Education Program’s Adult Treatment Panel III report (ATP III) or International Diabetes Federation (IDF) definitions [15] have reported metabolic syndrome prevalence of approximately 33% for schizophrenia [15] and 37% for bipolar disorder [2]. The ICD-9-CM coding did not allow for the clear identification of bipolar II disorder (falls under “bipolar other”), precluding the assessment of differences in the number of cardiovascular comorbidities and outcomes between bipolar I and bipolar II subtypes. Moreover, there was no control group of inpatients without serious mental illness. The analysis of each patient was limited to a single index hospitalization and 30 days post-discharge, rather than attempting to determine hospital costs or outcomes over a longer duration of follow-up. Readmission rates in this study may be an underestimate, as these data only include admissions to the hospitals in the Premier network, and the likelihood of readmission to hospitals has been reported to be as high as 20% elsewhere [22].

## Conclusions

In this large, retrospective, administrative database study, over 60% of patients with schizophrenia or bipolar disorder had at least one cardiometabolic comorbidity, and over 30% had two or more cardiometabolic comorbidities. For patients with schizophrenia, increasing cardiometabolic comorbidity burden had a significant impact on cost of index hospitalization, and 30-day readmission rates. For patients with bipolar disorder, increasing cardiometabolic comorbidity burden had a significant impact on length of stay, hospital mortality rates, cost of index hospitalization, and 30-day readmission rates. These results further underscore the need for improved detection and management of cardiometabolic risk factors in patients with schizophrenia or bipolar disorder across different clinical care settings. Further research is needed to better understand the long-term consequences of cardiometabolic comorbidity burden on patients with schizophrenia or bipolar disorder.

## Abbreviations

ATP III: National Cholesterol Education Program’s Adult Treatment Panel II
CCI: Charlson Comorbidity Index
GLMs: generalized linear models
ICD-9-CM: International Classification of Diseases, 9th Revision, Clinical Modification
IDF: International Diabetes Federation
US: United States
